# Supplementary material for: End Sequence Analysis Toolkit (ESAT) expands the extractable information from single-cell RNA-seq data
Source: Genome Res. 2016 Oct;26(10):1397–410. doi: 10.1101/gr.207902.116 (PMC5052061; doi:10.1101/gr.207902.116)
Supplement: Supplemental Material [file supp_gr.207902.116_Supp_Materials.tar.gz › ESAT-esat_v0.1/doc/html/classbroad_1_1core_1_1siphy_1_1_tree_scaler-members.html]

ESAT: Member List


ESAT


- Main Page
- Related Pages
- Packages
- Classes
- Files

- Class List
- Class Index
- Class Hierarchy
- Class Members

All Classes Namespaces Files Functions Variables Enumerator Pages

- broad
- core
- siphy
- TreeScaler

broad.core.siphy.TreeScaler Member List

This is the complete list of members for broad.core.siphy.TreeScaler, including all inherited members.

|  |  |  |
| --- | --- | --- |
| encodeAsMatrix() | broad.core.siphy.TreeScaler |  |
| fitPI(Phylogeny alnTree, int i) | broad.core.siphy.TreeScaler |  |
| getLogLikelihood(double omega, Phylogeny alnTree, int startPosition, int window) | broad.core.siphy.TreeScaler |  |
| getMinimumTreeLength() | broad.core.siphy.TreeScaler |  |
| getModel() | broad.core.siphy.TreeScaler |  |
| getTotalDistanceFromNode(PhylogenyNode n) | broad.core.siphy.TreeScaler |  |
| main(String[] args) | broad.core.siphy.TreeScaler | static |
| scaleRegion(List< String > ignoreList, GenomicAnnotation region) | broad.core.siphy.TreeScaler |  |
| scaleRegion(Annotation region) | broad.core.siphy.TreeScaler |  |
| scaleTree(int window, BufferedWriter bw, List< String > ignoreList, int overlap) | broad.core.siphy.TreeScaler |  |
| scaleTree(int window, List< String > ignoreList, int overlap) | broad.core.siphy.TreeScaler |  |
| scaleTreeWithSampling(int window, BufferedWriter bw, List< String > ignoreList, int numberToAverage) | broad.core.siphy.TreeScaler |  |
| setAlignment(String alnFile, String alnFileFormat) | broad.core.siphy.TreeScaler |  |
| setAlignment(MultipleAlignment alignment) | broad.core.siphy.TreeScaler |  |
| setMinimumTreeLength(double minimumTreeLength) | broad.core.siphy.TreeScaler |  |
| setModel(EvolutionaryModel model) | broad.core.siphy.TreeScaler |  |
| setNeutralModel(File modelFile) | broad.core.siphy.TreeScaler |  |
| slidePWM(PositionWeightMatrix pwm, double minSeedScore, short[] encodedRef, List< int[]> ungappedChunks, int numPermutations) | broad.core.siphy.TreeScaler |  |
| slidePWM(PositionWeightMatrix pwm, double minSeedScore, int numPermutations) | broad.core.siphy.TreeScaler |  |
| TreeScaler() | broad.core.siphy.TreeScaler |  |
| TreeScaler(Annotation region, File modelFile, String alnFile, String alnFileFormat) | broad.core.siphy.TreeScaler |  |
| USAGE | broad.core.siphy.TreeScaler | static |


---

Generated on Fri Aug 15 2014 15:10:57 for ESAT by  

 1.8.7
